# Incidence and risk factors for lung cancer among women in the women’s interagency HIV study (WIHS) and men in the multicenter AIDS cohort study (MACS)

**DOI:** 10.1186/1750-9378-7-S1-O24

**Published:** 2012-04-19

**Authors:** Nancy A Hessol, Otoniel Martinez-Maza, Alexandra Levine, Charles R Rinaldo, Joseph B Margolick, Mardge H Cohen, Lisa P Jacobson, Eric C Seaberg

**Affiliations:** 1 Departments of Clinical Pharmacy & Medicine, University of California, San Francisco, CA, USA; 2 Department of Obstetrics & Gynecology, University of California, Los Angeles, CA, USA; 3 City of Hope National Medical Center, Duarte, CA, and Keck School of Medicine, University of Southern California, Los Angeles, CA, USA; 4 Department of Pathology, University of Pittsburgh, PA, USA; 5 Department of Molecular Microbiology and Immunology, Bloomberg School of Public Health, Johns Hopkins University, Baltimore, MD, USA; 6 Department of Medicine, Stroger (formerly Cook County Hospital), and Rush University, Chicago, IL, USA; 7 Department of Epidemiology, Bloomberg School of Public Health, Johns Hopkins University, Baltimore, MD, USA

## Background

Studies have reported an increased incidence of lung cancer among people with HIV/AIDS compared to population estimates [1], but it is unclear whether this increase is due to HIV or to other lung cancer risk factors such as cigarette smoking. One study found that HIV-infected adults with preexisting lung disease displayed trends for increased lung cancer risk [2]. Another study of people with AIDS reported that individuals with recurrent pneumonia were at significantly higher lung cancer risk than those without [3]. Our aims were to determine the incidence and risk factors for lung cancer among participants in two longitudinal studies of HIV infection in United States women and men.

## Methods

Data from 3763 women in the WIHS and 6972 men in the MACS were analyzed and incidence rates (IR) per 100,000 person-years and rate ratios (IRR) were calculated.

## Results

We identified 44 lung cancer cases (33 HIV+ and 11 HIV-), 25 in the WIHS and 19 in the MACS, all with a history of smoking cigarettes. Among current and former smokers, the IR was significantly higher in the WIHS than in the MACS (WIHS IR=110.4 and MACS IR=35.8, p<0.001) but did not differ by HIV status. In multivariable analyses of the MACS participants, >30 pack-years of smoking (IRR=10.2) and a prior AIDS diagnosis (IRR=4.9) were significantly associated with an increased lung cancer rate. In multivariable analyses of the WIHS participants, age >49 (IRR=2.9 for 50-59; IRR=10.1 for 60+), Black race (IRR=4.6), >9 pack-years of smoking (IRR=14.7 for 10-30 pack-years; IRR=20.7 for >30 pack-years), and prior AIDS pneumonia (IRR=7.5) were significantly associated with and increased rate of lung cancer while more recent year of cohort enrollment (IRR=0.4 for 2001-2002) was associated with a lower rate.

## Conclusions

HIV infection was not associated with lung cancer in the WIHS and was no longer significant in the MACS after adjustment for a prior AIDS diagnosis. A prior diagnosis of AIDS pneumonia was a risk factor for lung cancer in the WIHS. Pack-years of smoking was a strong risk factors for lung cancer in both cohorts but was twice as strong in the WIHS. A better understanding of the effect of HIV on lung cancer is needed but cessation of smoking is an ideal goal for HIV-infected individuals.
